# 3D-Printable Knee Arthrometer: Development and Validation

**DOI:** 10.7759/cureus.74112

**Published:** 2024-11-20

**Authors:** Edmar Stieven Filho, Carolline P Nunes, Ayrton A Martins Neto, Isabela P Nascimento, Jose A Foggiatto, Fernando M Rosa, Paul A Milcent, Caue O Maia, Maisa S Namba, Lucas Santiago

**Affiliations:** 1 Orthopedics and Traumatology, Clinical Hospital of the Federal University of Paraná, Curitiba, BRA; 2 Orthopaedics and Traumatology, Workers' Hospital, Federal University of Paraná, Curitiba, BRA; 3 Medical Student, Federal University of Paraná, Curitiba, BRA; 4 Mechanical Engineering, Federal University of Technology – Paraná, Curitiba, BRA; 5 Orthopedics, Clinical Hospital of the Federal University of Paraná, Curitiba, BRA; 6 Orthopedics and Traumatology, Workers' Hospital, Curitiba, BRA; 7 Orthopedics, Clinics Hospital of Parana, Curitiba, BRA

**Keywords:** ‎3d printing, additive manufacturing, anterior cruciate ligament (acl), anterior cruciate ligament tear, arthrometer

## Abstract

Introduction

The anterior cruciate ligament is the most commonly injured ligament in the knee. Its injury is often evaluated with orthopedic tests during physical examination, but this turns out to be a subjective assessment. A knee arthrometer is a mechanical device developed in the 1970s to improve the diagnostic accuracy of anterior cruciate ligament injury. However, due to the high cost and low availability of the devices, they are rarely used in clinical practice and training. In this scenario, the objective of this study was to develop and validate a knee arthrometer to be fabricated by additive manufacturing (3D printing), thus creating a device that is easily accessible to all.

Methods

This experimental cross-sectional study involved the Orthopedic Skills Laboratory, Department of Surgery, at the Federal University of Paraná and 3D Printing Laboratory of the Professor Leide Parolin Marinoni Simulation Center of Parana Clinical Hospital, in partnership with the Engineering Department at the Federal University of Technology - Paraná, Brazil. Additionally, face validity was conducted at the Workers' Hospital and Parana Clinical Hospital. Ten orthopedic professionals with experience in the use of arthrometers were invited to assemble, evaluate the developed equipment, and answer a questionnaire regarding the assembly, appearance, and usability of the 3D arthrometer.

Results

At the end of the project, the arthrometer was successfully assembled from 3D-printed and unprinted parts, with an approximate cost of US$30 per device. All participants were able to assemble the 3D arthrometer with the instructions provided, and only one participant requested the instructor's assistance once during assembly. Regarding use, all participants were able to perform the arthrometry successfully and without the need for any assistance. Regarding face validity, we obtained a high degree of agreement among participants regarding the ease of assembly, good appearance, ease of use, and practicality of the equipment.

Conclusion

Using 3D printing technology to fabricate a knee arthrometer is feasible, resulting in an inexpensive and accessible tool.

## Introduction

The anterior cruciate ligament (ACL) is the most commonly studied ligament in the knee and one of the most researched of all the entire musculoskeletal system [1]. Furthermore, this ligament has a large socioeconomic impact, since ACL injury usually requires surgical treatment, with approximately 350,000 reconstructions per year in the United States, at an estimated cost of 1 billion dollars [2].

ACL ruptures are often evaluated with orthopedic tests, such as the Lachman test, the anterior drawer sign, and the pivot shift test, but they are subjective assessments [3]. The diagnostic sensitivity and specificity of the tests vary widely depending on the examiner’s experience, the patient’s body type, and the delay from injury to examination [4]. Although physical examination is the first step in the assessment of knee injuries, it has a low diagnostic value in the acute setting [5]. Furthermore, manual tests cannot quantify or detect minor changes in laxity (e.g., in the face of partial ACL tears) [6]. In a cadaveric study, Hole et al. found that the Lachman test was reliable only after sectioning at least 75% of the ligament [7].

To improve the accuracy of ACL injury diagnosis, the arthrometer was developed in the 1970s, which is a mechanical device capable of objectively measuring ligament laxity [8]. It is very useful to aid in the diagnosis of partial ligament ruptures and also to evaluate and monitor post-operative results [9]. Several arthrometer models have been developed covering a wide range of complexity and availability [10,11]. However, the use of arthrometers in the routine evaluation of patients with ACL injuries is still limited by the high cost, difficult use, and low availability of devices, as some models have been discontinued and are therefore difficult to find.

In this scenario, the purpose of this study was to develop and validate a knee arthrometer to be fabricated by additive manufacturing (3D printing), thus creating a device that is easily accessible to all orthopedists. 

## Materials and methods

This experimental cross-sectional study involved the Orthopedic Skills Laboratory, Department of Surgery, at the Federal University of Paraná and 3D Printing Laboratory of the Professor Leide Parolin Marinoni Simulation Center of Parana Clinical Hospital, in partnership with the Engineering Department at the Federal University of Technology - Paraná, Brazil. Additionally, face validity was conducted at the Workers' Hospital and Parana Clinical Hospital. The ethics committee approval number is 81340424.8.0000.5225.

**Figure 1:**
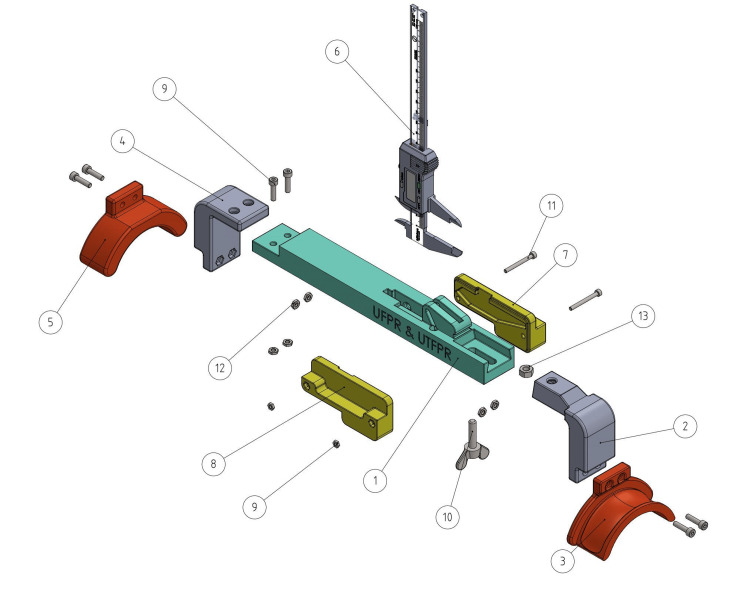
Scheme of parts and materials for assembling the 3D arthrometer. 1 - main bar (printed); 2 - proximal curve(printed); 3 - proximal arch (printed); 4 - distal curve (printed); 5 - distal arch (printed); 6 - caliper; 7 - left cover (printed); 8 - right cover (printed); 9 - M4 screw; 10 - butterfly M6 screw; 11 - M3 screw; 12 - M4 nut; 13 - M6 nut. Source: [12]

A 3D arthrometer was developed and modeled using SolidWorks® 3D CAD software (Dassault Systèmes, SolidWorks Corporation, Waltham, USA). The 3D printable components of the device were then fabricated by additive manufacturing (3D printing) with a Prusa i3 MK3S® printer using polyethylene terephthalate glycol (PETG) filament (Prusa, Prague, Czech Republic). The 3D printed and unprinted parts of the arthrometer are shown in Figure 1 and Table 1.

**Table 1:** Materials used and their respective costs

Pieces	Quantity	Cost
Caliper	1	US $9
Nylon Cable Ties 3,6mm	2	US $2
M4x16 screws - Hex Socket Cap Head	6	US $2.5
M4 nut	6	US $1.5
M3x30 screws - Hex Socket Cap Head	2	US $1
M3 nut	2	US $1
Butterfly M6 screw	1	US $2
M6 nut	1	US $1
Printed parts	7	US $10
Total Cost		US $30

To perform face validity, 10 orthopedic professionals with experience in the use of arthrometers were invited to assemble, evaluate the developed equipment, and answer a questionnaire regarding the assembly, appearance, and usability of the 3D arthrometer. Before assembly, participants watched a video with instructions, and during assembly, they had access to an instruction manual and a trained evaluator who could help if necessary. After assembly, participants watched a video with information on the use of the equipment and then had to perform arthrometry on a healthy participant. In the end, they were asked to answer a questionnaire with a Likert scale adapted for this study, with five questions formulated, which were graded on a spectrum of five descriptors between “Completely disagree” and “Completely agree”.

Descriptive and exploratory statistics were used, in addition to the degree of agreement of the items being assessed by the Content Validity Index (CVI), in which the sum of agreement of the items “Completely agree” and “Partially agree” marked by the participants was considered. An acceptable content validity index should be at least 0.80 and preferably greater than 0.90 [13].

Step-by-step assembly of the arthrometer

**Figure 2:**
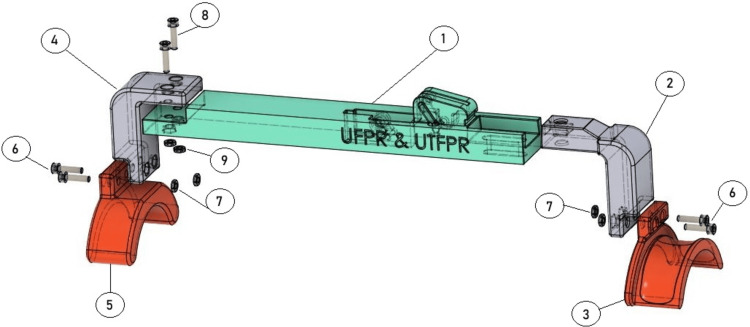
Assembling the device. Coupling the distal curve (5) to the distal arch (4), and the proximal arch (3) and proximal curve (2), with two M4x16 mm screws (6) and two M4 nuts (7);. Attaching the set, distal arch (4), and distal curve (2), to the main bar (1) of the arthrometer with two M4x16 screws (8) and two M4 nuts (9). Source: [12]

The first step is coupling the distal curve to the distal arch, with two M4x16 mm screws and two M4 nuts. The same procedure is performed for the proximal arch to the proximal curve. The set, distal arch, and distal curve are then coupled to the main bar of the arthrometer with two M4x16 screws and two M4 nuts (Figure 2).

**Figure 3:**
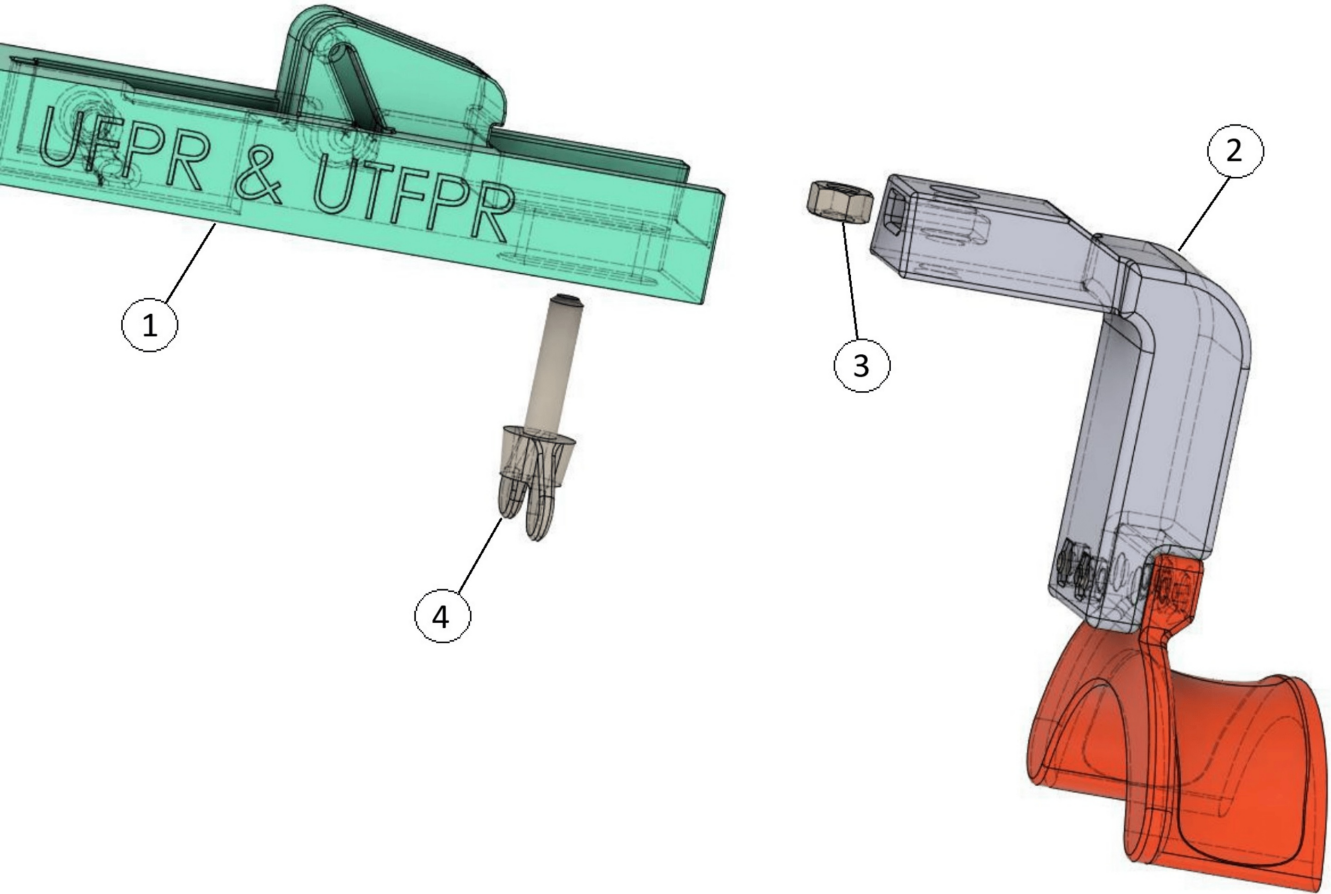
Insert an M6 nut (3) into the pre-countered hole of the proximal angle bracket (2), then tighten an M6x25 knurled thumb screw (4) from bottom to top to secure this section to the main bar (1). Source: [12]

Then, an M6 nut ​​is then placed inside the pre-countered hole of the proximal angle bracket, and an M6x25 knurled thumb screw is tightened from bottom to top to fix this region of the arthrometer to the main bar (Figure 3). The thumb screw allows changing the length of the arthrometer, being easily adaptable to the patient’s characteristics.

**Figure 4:**
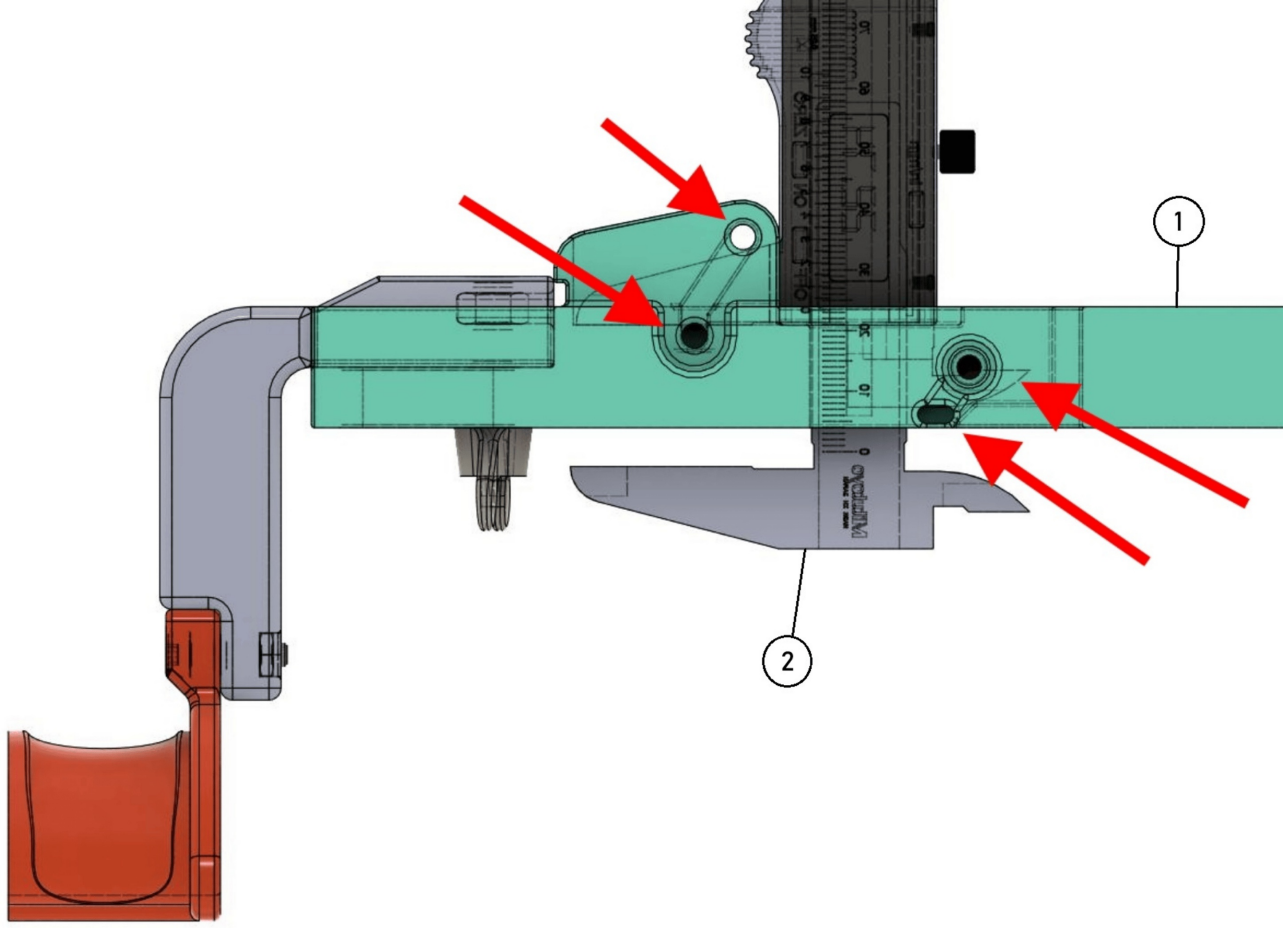
Insert the digital caliper (2) into the designated hole on the main bar's upper surface (1). Secure it with two nylon cable ties passed through pre-drilled holes (set in red), looping around the caliper jaws to fix it in place. Source: [12]

Subsequently, the measuring instrument (digital caliper) is inserted in the predetermined hole in the main bar on the upper surface of the device. The caliper is fixed to the main bar by passing two nylon cable ties through four pre-drilled holes in the main bar, looping the caliper jaws, and fixing the caliper to the main bar of the arthrometer (Figure 4).

**Figure 5:**
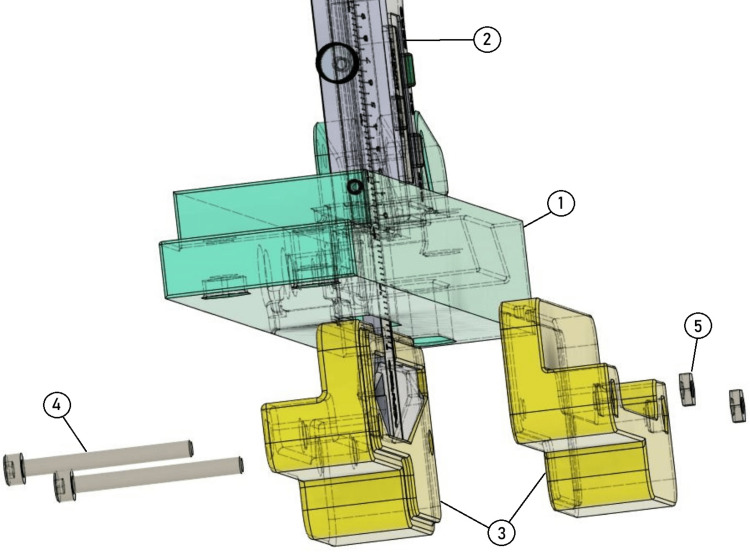
Fitting the caliper covers. To install the caliper cover, fit the cover pieces (3) around the caliper (2), below the main bar (1). To secure the cover in place use two M3 screws (4) and M3 nuts (5). Source: [12]

Lastly, two caliper covers (right and left) are then fixed to the caliper jaws with two M3x30 screws (Figure 5).

The entire process of assembling the arthrometer is demonstrated in a video attached as supplementary data of this paper (Video 1), as well as a video with instructions for using a 3D arthrometer (Video 2). These two videos were used as guides for research participants during the validation process.

**Video 1:** The entire process of assembling the arthrometer. Source: the author.

**Video 2:** How to use the 3D knee arthrometer. Source: the author.

## Results

**Figure 6:**
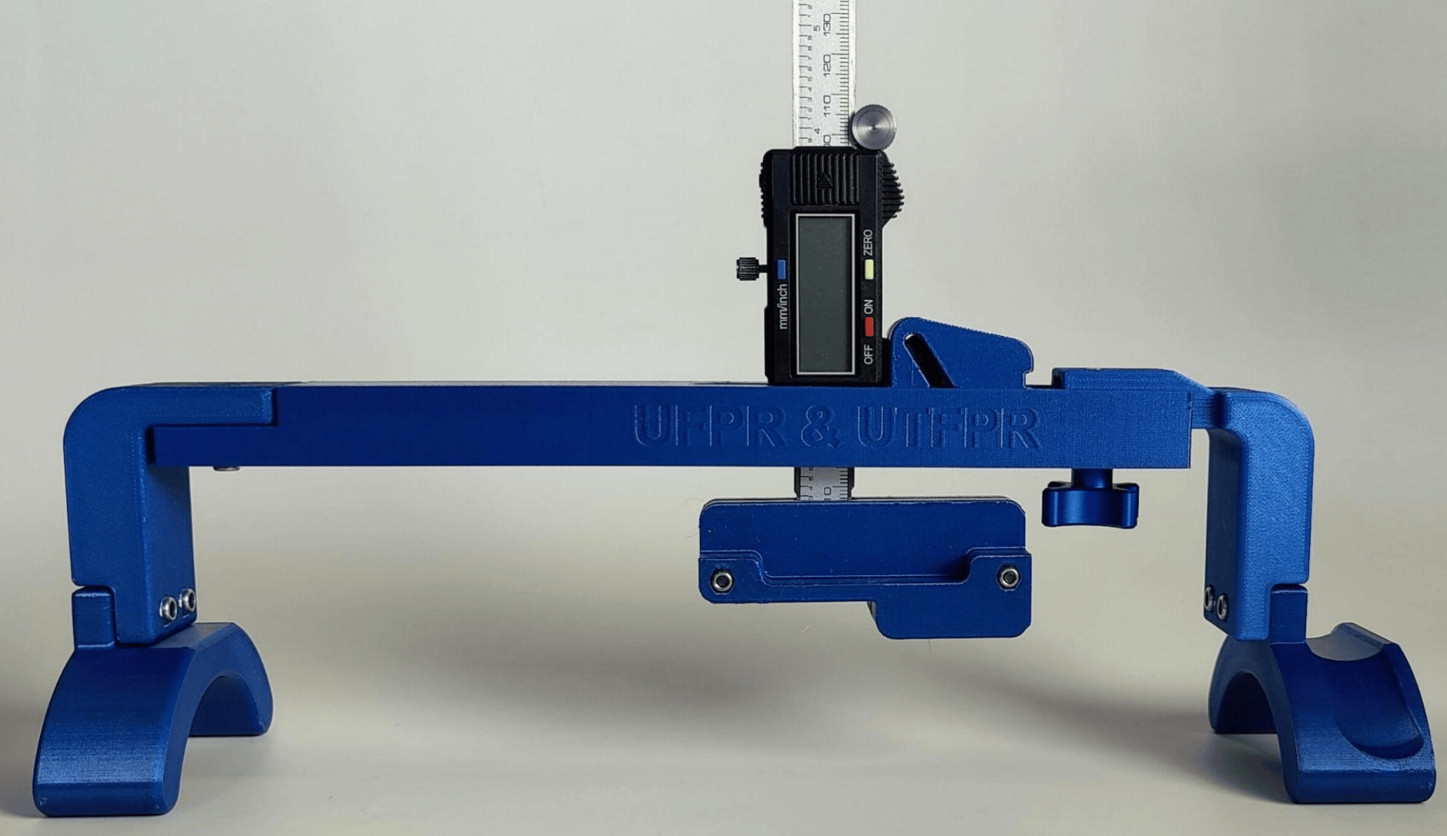
Arthrometer completely assembled and ready for use. Source: [12]

The arthrometer was successfully fabricated by 3D printing. Figure 6 shows the complete ready-to-use assembly of the device, which was accomplished at an approximate cost of US$30.

The participants evaluated were all men, orthopedists, and knee surgeons recognized by the SBCJ (Brazilian Society of Knee Surgery). All participants were able to assemble the 3D arthrometer with the instructions provided, and only one participant requested the instructor's assistance once during assembly. Regarding use, all participants were able to perform the arthrometry successfully and without the need for any assistance.

**Figure 7:**
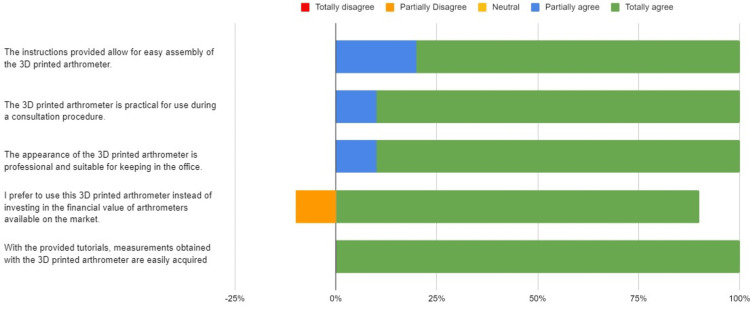
Graph with the questions and respective percentages of each answer.

As a result of the validation, in the graph in Figure 7, we have all the questions that were evaluated and their respective answers. In general, all the questions asked had a Content Validity Index above 0.9. The equipment obtained 100% agreement regarding the usability and ease of use of the equipment. Regarding assembly, 80% of the participants strongly agreed that the instructions provided allowed for easy assembly. Regarding the appearance of the 3D arthrometer and its practicality for use during a consultation procedure, 90% of the participants strongly agreed with the statements. The greatest disparity occurred in the question regarding the comparison of the 3D arthrometer with others available on the market, where 10% of participants disagreed with the statement, but the total IVC of the question remained at 0.9.

## Discussion

The rate of ACL injuries is increasing despite improved knowledge of ACL injury and prevention [1]. However, less than 10% of patients with an ACL injury have the diagnosis made by the first physician to see them [14]. The arthrometer was developed to improve the detection of ACL injuries and to quantify the results [5, 8, 15]. In the current study, we present an alternative arthrometer that is affordable, reproducible, easily portable, and easy to handle. We fabricated the arthrometer by additive manufacturing (3D printing) to obtain an affordable and high-strength tool.

The KT-1000 (MEDmetric Corp., San Diego, California) is the most widely used knee arthrometer and is currently considered the standard instrumented testing device for measuring anterior knee translation [16]. Van Eck et al. reported that arthrometer testing with the KT-1000 performed with maximum manual force had the highest sensitivity, specificity, accuracy, and positive predictive value for the diagnosis of ACL rupture [17]. Although considered the gold standard for assessing ACL injuries, the KT-1000 arthrometer was discontinued a few years ago, making it difficult to find and limiting access to the equipment. Furthermore, the device itself is considerably large and heavy, which makes it difficult to use during examination. Given these issues, several studies have attempted to develop a simpler and more portable device [4, 11]. Among them, the Rolimeter (Aircast Europa, Neubeuern, Germany) stands out, which is a simpler, more compact, and easier-to-handle device than the KT-1000 and is also easily portable [10, 11]. Several studies have compared the Rolimeter with the KT-1000, with a good correlation between the two devices; the main advantages of the Rolimeter are its low acquisition cost, simple learning curve, and lightness [11, 18-21]. However, the Rolimeter is no longer manufactured.

The device developed in this work can be used similarly to the Rolimeter to measure the anterior translation of the tibia in relation to the femur by applying maximum manual force. Because it is similar in size to the Rolimeter, it offers the same advantages: ease of handling, quick measurement procedure, and ease of transportation. We replaced the analog gauge with a digital caliper to simplify the assessment and improve precision [19]. A major advantage of our 3D printable arthrometer is its open-source license (Creative Commons 4.0 International License Attribution-ShareAlike) and its significantly lower cost compared to industrial arthrometers. Our device can be manufactured at a cost of approximately US$30, as shown in Table 1, while similar commercial arthrometers used to be sold for hundreds of euros. The 3D printable knee arthrometer project is already available for download and public use [12]. Furthermore, to facilitate access and use, the authors have made videos available on the assembly and use of the arthrometer. A “how to use” video proved necessary in our journey with the arthrometer, as we found that few professionals had experience using the device, precisely due to the difficulty in acquiring an arthrometer (Videos 1, 2).

Face validity aims to assess the degree to which the object in question appears effective in achieving its stated objectives [22]. Regarding this issue, the 3D arthrometer performed excellently in the questionnaire results. All questions asked had a Content Validity Index above 0.9. In other words, we obtained a high degree of agreement among participants regarding the usability, ease of assembly, appearance, and practicality of the equipment.

A simple measurement procedure and low acquisition cost are decisive for the successful use of a device in the doctor's daily routine and currently, orthopedic surgery training lacks arthrometers in daily practice. This device is essential both for the diagnosis of ACL injury and for research in the field of medical education. Ideally, each orthopedic surgeon in training should have an arthrometer available for individual use whenever necessary for progressive training and familiarity with the device. This ideal scenario is not currently attainable with the devices available in the market. We hope that the arthrometer model presented here will gain widespread use in clinical practice.

Limitations

The study has some limitations that should be considered. First, the sample size for face validation was limited, which may affect the generalizability of the findings to broader populations. Furthermore, the study did not include direct comparisons with existing arthrometers, which may limit the understanding of the device’s efficacy. Moreover, the lack of clinical validation also represents a significant limitation, as the arthrometer’s performance in real-world medical settings has not yet been fully evaluated. With these limitations in mind, we aim to conduct future studies to validate our 3D printable knee arthrometer using a simultaneous validation design, comparing it with other arthrometers and evaluating its clinical performance.

## Conclusions

Using 3D printing technology to manufacture a knee arthrometer is feasible, resulting in a cheap and accessible tool. During validation, all participants were able to assemble the 3D arthrometer with the instructions provided, and successfully perform arthrometry. Overall, participants expressed agreement on the device's usability, ease of assembly, the appearance of the 3D arthrometer and its practicality for use. We hope that this equipment will enable more orthopedic specialists to have easier access to their own personal arthrometers for individual use.
